# Comparison of Cardiac Activated Fibroblast Imaging and Magnetic Resonance Imaging in Patients with COVID-19-Related Myocarditis

**DOI:** 10.31083/j.rcm2505161

**Published:** 2024-05-09

**Authors:** Yao Su, Xin Liu, Boqia Xie, Bowen Zhang, Qi Yang, Min-Fu Yang

**Affiliations:** ^1^Department of Nuclear Medicine, Beijing Chaoyang Hospital, Capital Medical University, 100020 Beijing, China; ^2^Department of Radiology, Beijing Chaoyang Hospital, Capital Medical University, 100020 Beijing, China; ^3^Cardiac Center, Beijing Chaoyang Hospital, Capital Medical University, 100020 Beijing, China

**Keywords:** ^99m^Tc-HFAPi, SPECT/CT, CMR, COVID-19-related myocarditis, cardiac fibroblast activation

## Abstract

**Background:**

This study aimed to explore the association between cardiac fibroblast activation and cardiac magnetic resonance (CMR) imaging parameters in patients with myocarditis following infection with coronavirus 2019 (COVID-19).

**Methods:**

In this prospective study, four patients with COVID-19-related myocarditis underwent ^99m^Tc-labeled–hydrazinonicotinamide–fibroblast activation protein inhibitor-04 (^99m^Tc-HFAPi) single photon emission computed tomography/computed tomography (SPECT/CT) and CMR imaging. Segmental ^99m^Tc-HFAPi activity was quantified as the percentage of average segmental myocardial count × global left ventricular target-to-background ratio. T1/T2 values, extracellular volume (ECV), and late gadolinium enhancement (LGE) were analyzed by CMR. The consistency between myocardial ^99m^Tc-HFAPi activity and CMR parameters was explored.

**Results:**

In patients with myocarditis, the proportion of segments with abnormal ^99m^Tc-HFAPi activity was significantly higher than in those with abnormal LGE (81.25% vs. 60.93%, *p* = 0.011), abnormal T2 (81.25% vs. 50.00%, *p* < 0.001), and abnormal ECV (81.25% vs. 59.38%, *p* = 0.007); however, they were similar in those with abnormal native T1 (81.25% vs. 73.43%, *p* = 0.291). Meanwhile, ^99m^Tc-HFAPi imaging exhibited good consistency with native T1 (kappa = 0.69).

**Conclusions:**

Increased cardiac ^99m^Tc-HFAPi activity is present in COVID-19-related myocarditis, which is correlated with the native T1 values in CMR.

## 1. Introduction

The clinical syndromes associated with coronavirus disease 2019 (COVID-19) are caused by severe acute respiratory coronavirus 2 (SARS-CoV-2) [1]. It has been recognized that the main cofactors required for SARS-CoV-2 to enter human host cells are angiotensin-converting enzyme 2 and transmembrane protease serine 2, which can invade the myocardium and cause direct or indirect myocardial injuries. When myocardial injury occurs, troponin is released into the blood circulation, while the detection of troponin in peripheral blood can reflect the injury of cardiomyocytes. Therefore, myocardial troponin has been identified as a marker of myocardial injury [2, 3, 4, 5]. Although the most common viruses that cause myocarditis are enteroviruses, such as Coxsackie virus B3 and Parvovirus B19 [6], there is increasing evidence that the newly emerged SARS-CoV-2 can also cause myocarditis [7]. A study found that the prevalence of acute myocarditis was 2.4–4.1 cases per 1000 patients hospitalized with COVID-19 [7]. However, the exact pathophysiological mechanism of COVID-19-related myocarditis remains incompletely understood. Therefore, objective and accurate evaluation of COVID-19-related myocarditis may be imperative for the treatment and prognosis of patients.

Multiparametric cardiac magnetic resonance (CMR) has been used as a noninvasive method to detect myocardial function and tissue characteristics for the diagnosis and risk stratification of patients with suspected myocarditis. Recently, the Lake Louise criteria [8] for diagnosing acute myocarditis have been updated to incorporate new CMR quantitative imaging methods, such as T1 mapping [9], extracellular volume (ECV) (for detecting myocardial edema, extracellular space expansion, and fibrotic changes), and T2 mapping (for detecting more acute states of free water accumulation) [10, 11].

In patients with myocarditis, cardiac fibroblast activation is accompanied by inflammatory cell infiltration, proliferation, and resolution. Activated myofibroblasts migrate to injured tissue and promote fibrotic scar formation, although severe fibrosis can cause adverse ventricular remodeling and eventually progressive systolic and diastolic dysfunction [12]. Therefore, the detection of cardiac fibroblast activation in myocarditis is advantageous in understanding the extent, degree, and repair of myocardial injuries. However, there are no clinical studies on cardiac fibroblast activation in COVID-19-related myocarditis.

Fibroblast activation protein (FAP) is a type II transmembrane glycoprotein with collagenase activity, which is a specific marker for fibroblast activation. Recently, a radiolabeled FAP inhibitor (FAPI) has been extensively used for positron emission tomography/computed tomography (PET/CT) imaging in several cardiovascular diseases [13, 14]. However, whether FAPI imaging can be utilized in COVID-19-related myocarditis has yet to be established.

Therefore, we aimed to evaluate the feasibility of using ^99m^Tc-labeled–hydrazinonicotinamide-FAPI-04 (^99m^Tc-HFAPi) single photon emission computed tomography/CT (SPECT/CT) imaging in assessing COVID-19-related myocarditis and then exploring the relationship between cardiac fibroblast activation and CMR-based myocardial features.

## 2. Methods

### 2.1 Study Population

This prospective study was approved by the Institutional Ethics Committee of Beijing Chaoyang Hospital (2022-ke-535), and all patients provided written informed consent. Four patients with COVID-19-related myocarditis were recruited from December 2022 to February 2023. Inclusion criteria: (1) Confirmed to be infected with COVID-19 by positive results from nasopharyngeal swabs or elevated specific antibodies; (2) was diagnosed with myocarditis according to the 2018 Lake Louise consensus criteria [8], 17 (10–30) days after the clinical diagnosis of COVID-19 infection. Exclusion criteria: Patients with myocardial infarction and severe coronary artery disease. In this study, all patients had no history of hypertension and diabetes. For comparison, eight healthy volunteers were recruited to undergo ^99m^Tc-HFAPi SPECT/CT imaging as controls, and four healthy controls were selected from the magnetic resonance imaging database.

### 2.2 Normal Control Population

For comparison, eight healthy volunteers (two men; median [interquartile range (IQR)] age, 33 [25–39] years) were recruited to undergo ^99m^Tc-HFAPi SPECT/CT imaging as healthy controls. In addition, four healthy controls (one man; median [IQR] age, 32 [28–36] years) were selected from the magnetic resonance imaging database. The inclusion criteria were age- and sex-matched, no history of cardiovascular disease or cardiac abnormalities documented at cardiovascular examinations, and no history of malignancy.

### 2.3 ^99m^Tc-HFAPi Image Acquisition and Interpretation

^99m^Tc-HFAPi was radiolabeled, as previously described [15]. SPECT images were acquired 3 h after injection of 736.30 (626.78–783.29) MBq ^99m^Tc-HFAPi using a conventional SPECT camera equipped with a low-energy parallel hole collimator (Infinia Hawkeye 4, GE, USA). Electrocardiogram-gated myocardial SPECT imaging was acquired using the following acquisition settings: Matrix (64 × 64) and condition (zoom 1.0, 20 s/step, from 45° right anterior oblique view to 45° left posterior oblique at 6° intervals). CT parameters were as follows: 120 kV, 1 mA, pitch of 1.9, and slice thickness of 5 mm.

SPECT images were reconstructed by three-dimensional ordered-subset expectation maximization (10 subsets, 2 iterations), and CT images were reconstructed by standard reconstruction to obtain integrated SPECT and CT images.

**Fig. 1.**
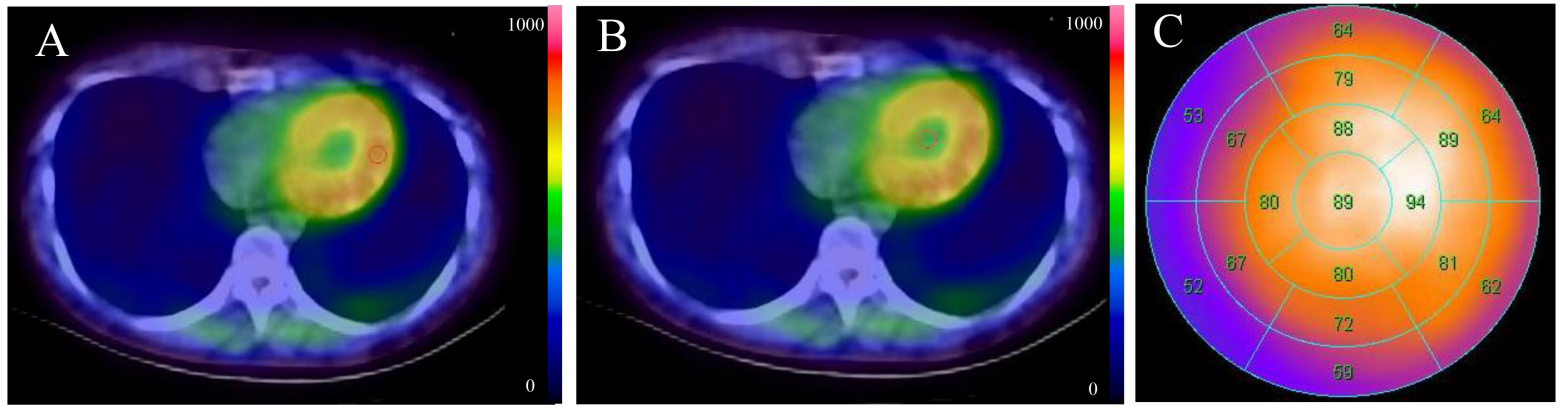
**A schematic diagram of ^𝟗𝟗𝐦^Tc-HFAPi image measurement**. Representative axial single photon emission computed tomography (SPECT/CT) images measuring regions of interest (ROIs) in the left ventricular myocardium with visual uptake (A) and left ventricular cavity (B). (C) is a representative polar plot. The numbers shown on the 17-segmental were automatically calculated using QPS software. The number divided by 100% was expressed as the percentage of the average segment count. ^99m^Tc-HFAPi, ^99m^Tc-labeled–hydrazinonicotinamide–fibroblast activation protein inhibitor-4.

Two nuclear physicians (YS and MFY) who were blinded to the clinical information and CMR data independently evaluated the ^99m^Tc-HFAPi images. Disagreements were resolved by consensus. A circular region of interest (semidiameter of 5 mm) was carefully placed on the area exhibiting the highest visual uptake of ^99m^Tc-HFAPi on each SPECT and CT registered transaxial image, and the maximum count was recorded to represent the left ventricular (LV) ^99m^Tc-HFAPi uptake. To obtain the background value of ^99m^Tc-HFAPi uptake, a region of interest of the same size was placed on the LV cavity, and the mean count was recorded (Fig. 1A,B). Then, a global LV target-to-background ratio (TBR) was calculated by dividing the maximal myocardial count by the mean count of the LV cavity.

The raw data in the ^99m^Tc-HFAPi images were processed using QPS software (version 3.1; Cedars-Sinai Medical Center, California, USA), and the polar plot was generated. Segmental ^99m^Tc-HFAPi uptake was automatically calculated using QPS software based on the American Heart Association 17-segment model and expressed as the percentage of the average segmental count (Fig. 1C). The product of this percentage and global LV TBR was calculated and defined as segmental ^99m^Tc-HFAPi activity. The ^99m^Tc-HFAPi-positive segment was defined as two standard deviations above the mean global TBR of the controls. The apical segment was excluded from the intersegment comparison analysis of ^99m^Tc-HFAPi activity and CMR.

### 2.4 CMR Image Acquisition and Interpretation

CMR was performed using a 3.0 T scanner (Prisma, Siemens Healthineers, Erlangen, Germany). Pre- and post-contrast T1 mapping, T2 mapping, late gadolinium enhancement (LGE), and cine were acquired.

CMR images were analyzed using commercial software (Cvi42, version 5.12.2, Circle Cardiovascular Imaging Inc., Calgary, Canada) by two experienced CMR operators (XL and QY) who were blinded to both the clinical and ^99m^Tc-HFAPi data. Disagreements were resolved by consensus. The T1 and T2 values and ECV are the average of all three slices of the myocardium. ECV was measured using a standard formula ECV = (1 – hematocrit) × (1/${\mathrm{T1}}_{\text{myopost}}$ – 1/${\mathrm{T1}}_{\text{myopre}}$)/(1/${\mathrm{T1}}_{\text{bloodpost}}$ – 1/${\mathrm{T1}}_{\text{bloodnative}}$) and expressed as ECV%. The apex segment was not included in the comparative analysis between ^99m^Tc-HFAPi and CMR images performed on a segment-by-segment basis.

Abnormal T1/T2 values and ECV were defined as two standard deviations above the mean of the reference values per the Society for Cardiovascular Magnetic Resonance recommendations [16]. Scars were detected and measured on the LGE images by setting a threshold of five standard deviations above the mean signal intensity of the unaffected myocardium. The indices of cardiac function were automatically mapped based on the left ventricular endocardial and epicardial contours at the diastole and systole ends in a short-axis cine sequence.

### 2.5 Statistical Analysis 

Statistical analysis was performed using software (SPSS Statistics version 26.0, Chicago, IL, USA). Continuous variables were expressed as medians with IQR, and categorical variables were expressed as frequencies or percentages. The kappa consistency test was used to explore the relationship between ^99m^Tc-HFAPi activity and CMR parameters. The variables of ^99m^Tc-HFAPi uptake and CMR results were compared using the chi-square test. *p* values of less than 0.05 were considered statistically significant.

## 3. Results

### 3.1 Patients’ Characteristics

Table 1 provides the detailed characteristics of the four patients with myocarditis. The median [IQR] age was 33 [21–38] years. The median [IQR] time from a positive SARS-CoV-2 test to CMR was 17 [10–30] days. The median [IQR] interval between CMR and ^99m^Tc-HFAPi SPECT/CT imaging was 4 [1–7] days. Two patients (50.00%) presented with dyspnea and palpitations, three patients (75.00%) had chest discomfort and heart failure, and all patients developed symptoms of fever.

**Table 1.** **Patients’ characteristics**.

Patient No.	Male	Age (years)	Dyspnea	Chest discomfort	Palpitations	Fevers	Heart failure	Peak cTnI (ng/mL) (0–0.04)	Peak CK (U/L) (50–310)	Peak CKMB (ng/mL) (≤5)	Peak BNP (pg/mL) (0–100)	Positive SARS-CoV-2 test to MRI (days)	MRI to SPECT (days)	TBR	LVEF (%) (56–78)	EDV (mL/$m^{2}$) (47–92)	ESV (mL/$m^{2}$) (13–30)	LV mass (g) (118–238)	Regional wall motion abnormality	ARNI	β-blockers	TMZ
1	No	39	Yes	Yes	No	Yes	Yes	2.73	280	5.0	519	19	5	3.30	52.6	50.4	23.9	107.1	Septum	No	No	Yes
2	Yes	30	No	No	Yes	Yes	Yes	9.07	95	24.3	34	9	2	4.25	65.6	84.3	29.0	146.2	None	No	Yes	Yes
3	No	36	Yes	Yes	No	Yes	Yes	0.10	55	0.2	113	34	7	4.67	53.0	95.0	45.4	139.2	Septum and inferior	Yes	Yes	Yes
4	Yes	18	No	Yes	No	Yes	No	3.50	125	4.4	191	14	0	2.47	54.1	78.0	35.8	121.2	None	No	No	Yes

cTnI, cardiac troponin I; CK, creatine kinase; CKMB, creatine kinase isoenzymes; BNP, brain natriuretic peptide; MRI, magnetic resonance imaging; SPECT, single photon emission computed tomography; TBR, target-to-background ratio; SARS-CoV-2, severe acute respiratory coronavirus 2; LVEF, left ventricular ejection fraction; EDV, end-diastolic volume; ESV, end-systolic volume; ARNI, angiotensin receptor-neprilysin inhibitor; TMZ, Trimetazidine; LV, left ventricular.

### 3.2 Characteristics of Cardiac Fibroblast Activation 

**Fig. 2.**
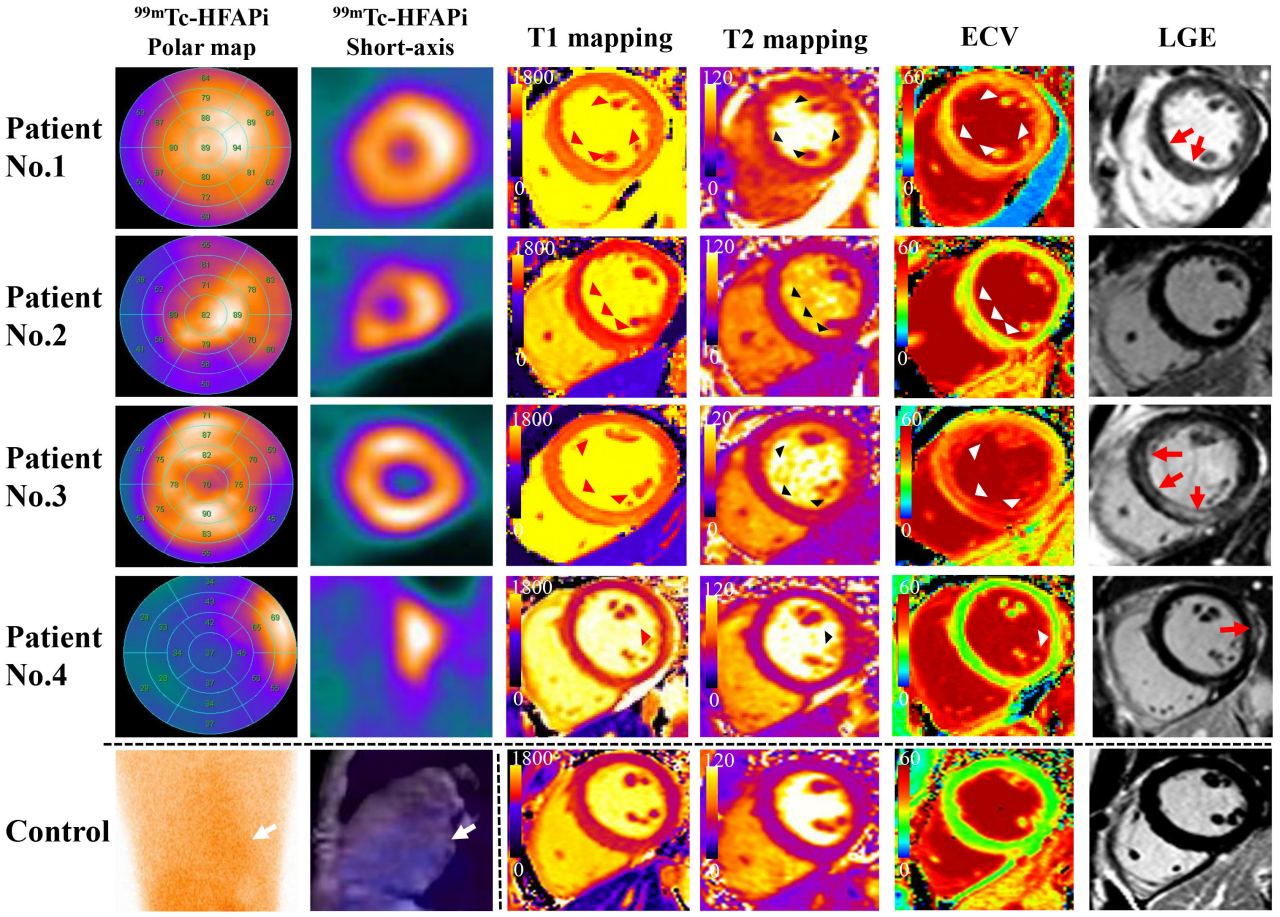
**Detailed images of the four patients and a healthy control**. Polar maps (first column) and the selected short-axis images of ^99m^Tc-HFAPi (second column), T1 mapping (third column), T2 mapping (fourth column), ECV (fifth column), and LGE (sixth column) of four patients. Intense diffuse (Patient No. 1–No. 3) or focal (Patient No. 4) ^99m^Tc-HFAPi uptake was observed in the LV, along with increased native T1 values (1380.6 [1280.4–1464.9] ms) (red triangle), increased T2 values (48.5 [43.0–53.4] ms) (black triangle), increased ECV (36.6% [29.6–41.6]) (white triangle), and LGE (red arrow). The segment with increased ^99m^Tc-HFAPi uptake matched with abnormal LGE in Patient No. 4. The fifth line is the ^99m^Tc-HFAPi plane image (first column), sagittal tomography image (second column) of ^99m^Tc-HFAPi health control, and T1 mapping (third column), T2 mapping (fourth column), ECV (fifth column), and LGE (sixth column) of CMR health control. No ^99m^Tc-HFAPi uptake was observed in the LV (white arrow). ^99m^Tc-HFAPi, ^99m^Tc-labeled–hydrazinonicotinamide–fibroblast activation protein inhibitor-4; SA, short axis; ECV, extracellular volume; LGE, late gadolinium enhancement; LV, left ventricular; CMR, cardiac magnetic resonance.

**Fig. 3.**
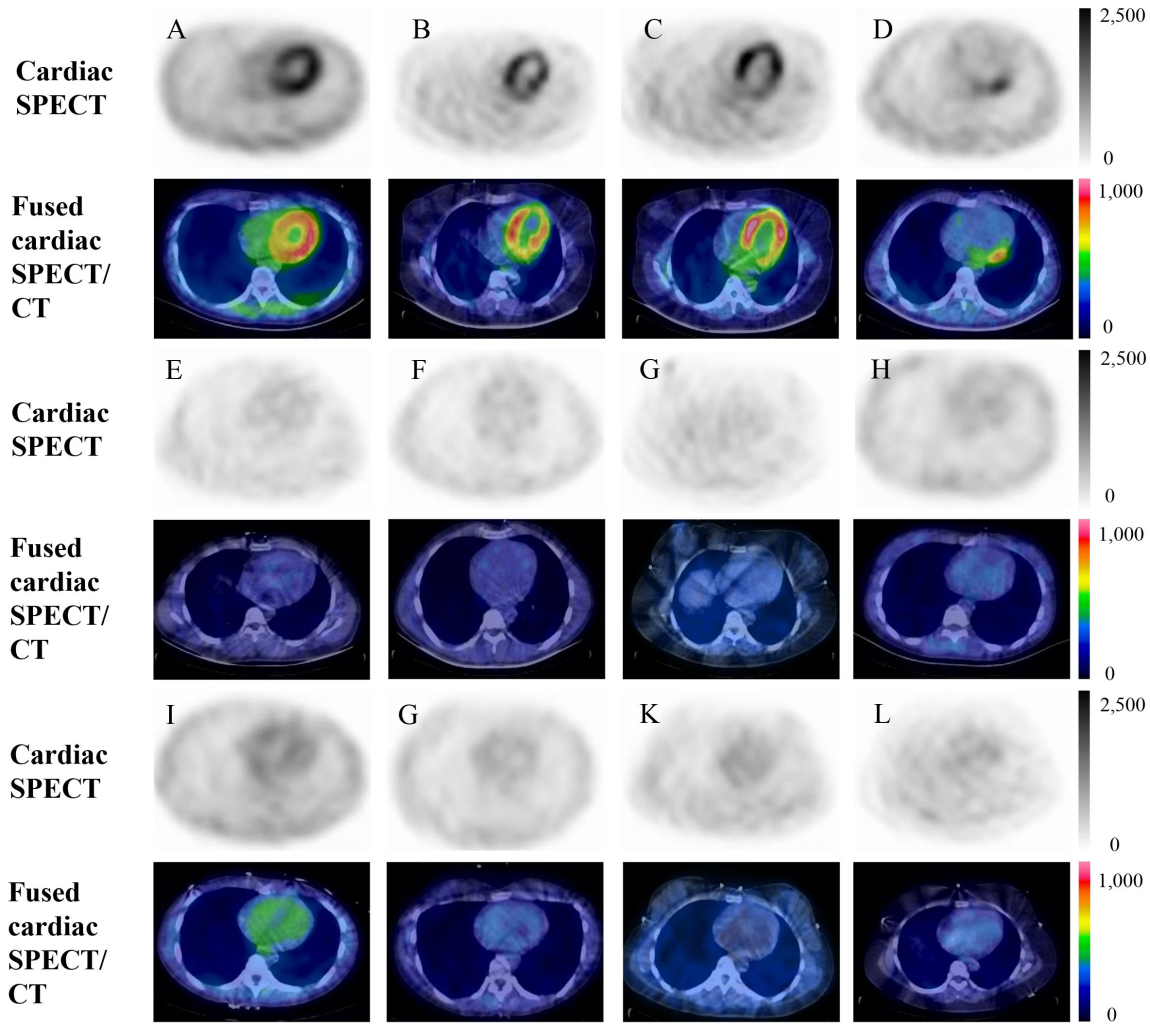
**Axial ^99m^Tc-HFAPi SPECT/CT imaging of the heart in COVID-19–myocarditis patients (Patient No.1–4, A–D) and eight control subjects (without cardiac disease, E–L)**. Each person has two images, including cardiac SPECT, with the fused cardiac SPECT/CT underneath. COVID-19–myocarditis patients demonstrate different patterns of myocardial ^99m^Tc-HFAPi uptake, categorized as diffuse uptake (Patient No.1–3, A–C) and focal uptake (Patient No.4, D), whereas no uptake is observed in the myocardium of normal controls. ^99m^Tc-HFAPi, ^99m^Tc-labeled–hydrazinonicotinamide–fibroblast activation protein inhibitor-4; SPECT, single photon emission computed tomography; COVID-19, coronavirus disease 2019; CT, computed tomography.

^99m^Tc-HFAPi and CMR images of four patients with myocarditis are presented in Fig. 2. All of them had diffuse (3/4, 75.00%) or focal (1/4, 25.00%) ^99m^Tc-HFAPi uptake in the LV, whereas no visible uptake was observed in the healthy controls, as shown in Fig. 3.

### 3.3 Relationship between Cardiac Fibroblast Activation and CMR Features

In healthy controls, the average native T1 value was 1215.03 ± 38.28 ms, ECV was 28.23 ± 2.31, and the T2 value was 40.51 ± 3.89 ms. Of the 64 segments in four patients with myocarditis, 47 (73.44%) were abnormal in native T1 mapping, 38 (59.38%) had ECV abnormalities, and 32 (50.00%) were abnormal in native T2 mapping.

**Fig. 4.**
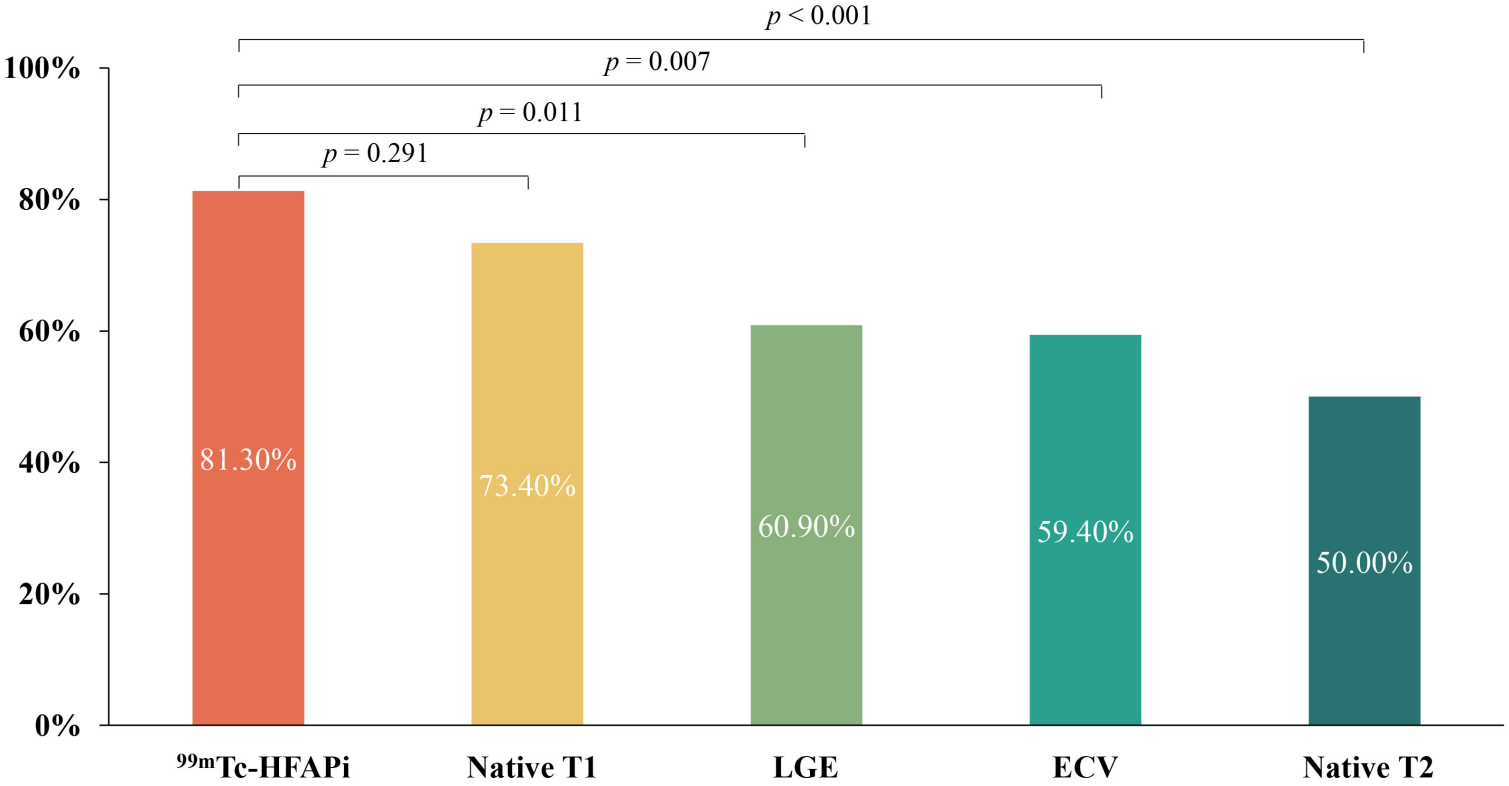
**The proportion of segments with increased ^99m^Tc-HFAPi uptake and abnormal CMR parameters**. Statistical significance was assessed by the chi-square test. *p* values of less than 0.05 were considered statistically significant. ^99m^Tc-HFAPi, ^99m^Tc-labeled–hydrazinonicotinamide–fibroblast activation protein inhibitor-4; CMR, cardiac magnetic resonance; LGE, late gadolinium enhancement; ECV, extracellular volume.

^99m^Tc-HFAPi imaging had good consistency with native T1 (kappa = 0.69) but only fair-to-moderate consistency with ECV (kappa = 0.50), LGE (kappa = 0.46), and native T2 (kappa = 0.31) (Table 2).

In patients with myocarditis, the proportion of segments with abnormal ^99m^Tc-HFAPi activity was significantly higher than in those with abnormal LGE (81.25% vs. 60.93%, *p* = 0.011), abnormal T2 (81.25% vs. 50.00%, *p* < 0.001), and abnormal ECV (81.25% vs. 59.38%, *p* = 0.007); however, it was similar to those with abnormal native T1 (81.25% vs. 73.44%, *p* = 0.291) (Fig. 4).

**Table 2.** **Consistency analysis of cardiac fibroblast activation and CMR parameters**.

			^99m^Tc-HFAPi activity			Kappa	*p* value
			Positive	Negative	All		
CMR parameters	Native T1	Abnormal	46	6	52	0.691	<0.001
		Normal	1	11	12		
		All	47	17	64		
	Native T2	Abnormal	31	21	52	0.313	<0.001
		Normal	1	11	12		
		All	32	32	64		
	ECV	Abnormal	38	14	52	0.504	<0.001
		Normal	12	0	12		
		All	50	14	64		
	LGE	Abnormal	38	14	52	0.457	<0.001
		Normal	1	11	12		
		All	39	25	64		

^99m^Tc-HFAPi, ^99m^Tc-labeled–hydrazinonicotinamide–fibroblast activation protein inhibitor-4; ECV, extracellular volume; LGE, late gadolinium enhancement; CMR, cardiac magnetic resonance. The kappa scores were interpreted as follows: <0.20, poor agreement; 0.21–0.40, fair agreement; 0.41–0.60, moderate agreement; 0.61–0.80, good agreement; and 0.81–1, excellent agreement.

## 4. Discussion

In this study, we found that 3/4 (75.00%) of patients with myocarditis had heart failure, and none had myocardial infarction, pericarditis, etc. We speculate that this may be related to endothelial dysfunction and hypercoagulability caused by SARS-COV-2 [17]. We also found cardiac fibroblast activation in all patients with COVID-19-related myocarditis. The area of fibroblast activation was larger than the area of pathophysiological abnormality identified by CMR [13, 14]. The main pathophysiological mechanisms of myocarditis include early inflammatory cell infiltration, cardiomyocyte edema, injury, and necrosis [18]. Studies have found that in the acute and recovery periods of the inflammatory response, many cytokines, such as interleukin-4 and transforming growth factor-β, can stimulate the proliferation and differentiation of fibroblast, thereby causing ventricular remodeling and cardiac dysfunction [19]. In our study, fibroblast activation was observed in all patients with COVID-19-related myocarditis. Thus, fibroblast activation is an important pathophysiological and functional feature of COVID-19-related myocarditis and is closely related to inflammation.

CMR has high sensitivity and specificity in identifying the pathological features of myocardial inflammation, edema, and fibrosis [8]. However, myocardial fibrosis detected by CMR mainly depends on the deposition of the extracellular matrix (ECM) in the interstitial space, which cannot be reversed once ECM is formed. ^99m^Tc-HFAPi SPECT/CT imaging can specifically detect activated fibroblasts, and ECM has not been synthesized at this stage. Therefore, ^99m^Tc-HFAPi SPECT/CT imaging could theoretically detect the process of myocardial fibrosis earlier than CMR tissue characterization techniques. Our study indicated that fibroblast activation was significantly correlated with native T1 values. We hypothesized that T1 values reflect comprehensive changes in the extracellular matrix, and both edema and fibrosis deposition in the extracellular matrix could lead to abnormal T1 values. Other CMR markers demonstrated distinct characteristics of abnormal tissue, such as the T2 values indicating edema and LGE indicating fibrosis. Both edema and inflammation trigger fibroblast activation [20], making increased myocardial ^99m^Tc-HFAPi uptake more relevant to T1 values. Our study also showed that fibroblast activation was weakly correlated with LGE. This result may be because LGE diagnoses myocardial fibrosis by detecting collagen deposition in the extracellular space resulting from fibroblast activation. However, FAP is a type II transmembrane glycoprotein with collagenase activity, which is a promising target for fibrosis imaging and a marker for fibroblast activation.

Although CMR is a promising technology in diagnosing myocarditis, more imaging techniques should be explored to reveal the myocarditis mechanism. Previous studies had used FAPI PET/CT to detect myocardial changes caused by immune checkpoint inhibitor-related myocarditis, although no relationship was identified between the CMR results and FAPI accumulation [21]. Here, for the first time, we used ^99m^Tc-HFAPi SPECT/CT to detect COVID-19-related myocarditis and found that ^99m^Tc-HFAPi activity correlated well with the T1 values of CMR. We hypothesized that this result may be because inflammatory factors can simultaneously cause myocardial edema and fibroblast activation.

## 5. Limitations

This work had some limitations. First, because of the rare manifestation of COVID-19-related myocarditis, we could only enroll four patients and did not obtain the cardiac pathology of each patient. Second, we did not analyze the relationship between ^99m^Tc-HFAPi activity and myocardial enzymes. Third, the four patients had different patterns of ^99m^Tc-HFAPi uptake, so in future studies, it is necessary to enroll more patients to analyze the type of ^99m^Tc-HFAPi uptake and explore the reasons for the different uptake patterns. Finally, in the present study, our focus was solely on the feasibility of ^99m^Tc-HFAPi imaging in evaluating early fibrosis in patients with COVID-19-related myocarditis, while a long-term follow-up after COVID-19 was not available. However, we are currently following up with patients with COVID-19-related myocarditis, and subsequent studies with larger samples will be needed to focus on whether ^99m^Tc-HFAPi imaging can provide additional prognostic value in patients with COVID-19-related myocarditis.

## 6. Conclusions

This study suggests that fibroblast activation can occur in areas of significant myocardial edema and increased extracellular space identified by CMR imaging, indicating that ^99m^Tc-HFAPi SPECT/CT imaging can detect myocarditis.

## Data Availability

The datasets used or analysed during the current study are available from the corresponding author on reasonable request.
